# Extracting Kondo temperature of strongly-correlated systems from the inverse local magnetic susceptibility

**DOI:** 10.1038/s41467-021-21641-2

**Published:** 2021-03-04

**Authors:** A. A. Katanin

**Affiliations:** 1 Center for Photonics and 2D Materials, Moscow Institute of Physics and Technology, Dolgoprudny, Moscow Region Russia; 2 M. N. Mikheev Institute of Metal Physics, Ekaterinburg, Russia

**Keywords:** Computational methods, Electronic properties and materials, Magnetic properties and materials

**Arising from** X. Deng et al. *Nature Communications* 10.1038/s41467-019-10257-2 (2019)

The temperature scales of screening of local magnetic and orbital moments are important characteristics of strongly correlated substances. In a recent paper, Deng et al.^1^ using dynamic mean-field theory (DMFT) have identified temperature scales of the onset of screening in orbital and spin channels in some correlated metals from the deviation of temperature dependence of local susceptibility from the Curie law. We argue that the scales obtained this way are in fact much larger than the corresponding Kondo temperatures, and, therefore, do not characterize the screening process. By reanalyzing the results of this paper we find the characteristic (Kondo) temperatures for screening in the spin channel *T*_K_ ≈ 100 K for V_2_O_3_ and *T*_K_ ≈ 350 K for Sr_2_RuO_4_, which are almost an order of magnitude smaller than those for the onset of the screening estimated in the paper (1000 K and 2300 K, respectively); for V_2_O_3_ the obtained temperature scale *T*_K_ is therefore comparable to the temperature of completion of the screening, *T*^cmp^ ~ 25 K, which shows that the screening in this material can be described in terms of a single temperature scale.

Deng et al.^1^ have performed a detailed analysis of the temperature dependence of orbital and magnetic local susceptibilities of two strongly correlated materials, Sr_2_RuO_4_ and V_2_O_3_ within DMFT^2–4^. At high temperatures, the susceptibilities obey the Curie law, *χ*(*T*) ~ 1/*T*. The temperatures *T*^ons^ of the onset of screening of spin- and orbital local moments are obtained from the deviation of *Tχ*(*T*) from a constant value. Corresponding temperature scales *T*^ons^ are found to be much larger than the scales, corresponding to the completed screening (onset of the Fermi-liquid behavior) *T*^cmp^ ~ 25 K.

In the following, we argue however that the temperatures *T*^ons^, obtained by the authors, do not correspond to the temperature scales of the spin screening. Indeed, instead of considering the quantity *Tχ*(*T*), we plot inverse spin susceptibility *χ*^−1^(*T*) for both considered compounds, Sr_2_RuO_4_ and V_2_O_3_ on the basis of the data of the paper (see Fig. 1). For Sr_2_RuO_4_ (see Fig. 1a) we do not find any peculiarity at the onset temperature *T*^ons^ = 2300 K suggested by the authors. Instead, at all considered temperatures the local susceptibility follows the Curie–Weiss law:

1 $$\chi (T)=\frac{C}{T+\theta }$$

with a positive temperature *θ* ≈ 500K (in agreement with the earlier result of ref. ^5^ and experimental data^6^). Following Wilson’s result for the local spin *S* = 1/2 Kondo problem^7–9^, the temperature $\theta \simeq \sqrt{2}{T}_{{\rm{K}}}$ yields the temperature scale of screening of the local moment (Kondo temperature) *T*_K_. Since the dependence *χ*(*T*)/*χ*(0) is almost universal for different *S*values ^10^, the abovementioned relation between *θ* and *T*_K_ is also expected to hold approximately for arbitrary local spin *S*. Therefore, for Sr_2_RuO_4_ we find the temperature scale of spin screening *T*_K_ ≈ 350 K, which is much smaller than *T*^ons^, obtained by the authors. We also note that very similar linear dependence of the inverse susceptibility is observed in the other Hund metals: *α*-iron (*T*_K_ = 50 K for density–density interaction and *T*_K_ ≈ 320 K for Kanamori interaction)^11–13^, *γ*-iron (*T*_K_ ~ 700 K)^14^, nickel (*T*_K_ ~ 850 K)^13^, etc.

**Fig. 1:**
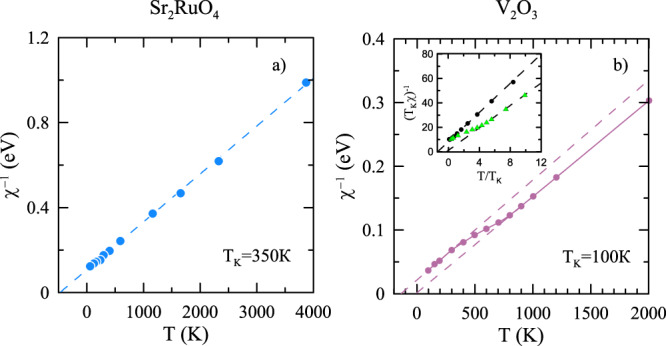
Temperature dependences of inverse local susceptibilities *χ*^−1^ (*T*). The inverse susceptibility, calculated from the data of ref. ^1^ for Sr_2_RuO_4_ is shown on the plot (**a**) and that for V_2_O_3_ is shown on the plot (**b**). The inset in (**b**) shows inverse spin susceptibility of the single-band half-filled Hubbard model on the square lattice with nearest-neighbor hopping *t* and on-site Coulomb repulsion *U* = 9*t* (triangles) in comparison to the inverse spin susceptibility of the Kondo model^7^ (circles); the Kondo temperature *T*_K_ = 0.032*t* of the Hubbard model is extracted from the fit of the low-temperature part of the susceptibility to the Kondo model. Dashed lines show linear fits to the data.

For V_2_O_3_ the situation is more complex, since the inverse susceptibility shows at *T* ~ 600 K a crossover (see Fig. 1b) from the Curie behavior (*θ* ≈ 0) to Curie–Weiss behavior with *θ* ≈ 150 K. This crossover, however, is likely not related to the screening, but reflects passing from a crossover regime to metallic one in the vicinity of Mott metal–insulator transition for this compound^15–17^. To confirm this viewpoint, we present in the inset of Fig. 1b the temperature dependence of the inverse local spin susceptibility of the single-band half-filled Hubbard model on the square lattice with nearest-neighbor hopping *t* (on-site Coulomb repulsion *U* = 9*t* is in the vicinity of Mott transition), showing that this dependence is qualitatively similar to the one, obtained for V_2_O_3_. Therefore, the screening scale of local magnetic moments in V_2_O_3_ is again given by the Kondo temperature ${T}_{{\rm{K}}}=\theta /\sqrt{2}\approx 100$ K, extracted from the low-temperature part of the local susceptibility in the paramagnetic phase. The latter value is also much smaller than obtained by the authors and has the same order of magnitude as the temperature, at which the screening is completed, *T*^cmp^ ~ 25 K. This makes it reasonable to describe spin screening in V_2_O_3_ in terms of a single energy (or temperature) scale, as it should be for a screening process of a single impurity site, considered in DMFT. We note that rather large Weiss temperature of local spin susceptibility of V_2_O_3_ (~600 K), observed experimentally in nuclear magnetic resonance studies^18,19^ in the temperature range *T* > 150 K may be related to the impact of strong antiferromagnetic correlations on local susceptibility, which is absent in paramagnetic DMFT solution.

The observation that for V_2_O_3_ the Kondo temperature *T*_K_ ~ *T*^cmp^ is in contrast to the above-described situation in Sr_2_RuO_4_, where *T*_K_ ≫ *T*^cmp^ ~ 25 K. We note that such an inequality is also fulfilled for nickel^13^, and in that case this was attributed to underscreened Kondo effect, since the Fermi level of nickel is close to the upper edge of the band. The origin of the strong difference between Kondo temperature and the temperature, corresponding to the completion of the screening in Sr_2_RuO_4_, requires further studies and clarification.

## Data Availability

The DMFT data for susceptibility of Sr_2_RuO_4_ and V_2_O_3_, analyzed here, are taken from ref. ^1^. The data on the single-band model are available within the present paper.
